# Rare Mucinous Adenocarcinoma of the Appendix Undergoing Multiple Recurrent Surgical Interventions

**DOI:** 10.7759/cureus.33294

**Published:** 2023-01-03

**Authors:** Usman Ilyas, Zaryab Umar, Amee M Pansuriya, Abrahim Mahmood, Muhammad Haseeb ul Rasool, Zamaraq Bhatti

**Affiliations:** 1 Internal Medicine, Icahn School of Medicine at Mount Sinai, Queens Hospital Center, Queens, USA; 2 Internal Medicine, New York Institute of Technology College of Osteopathic Medicine, Old Westbury, USA; 3 Medicine, Icahn School of Medicine at Mount Sinai, Queens Hospital Center, Queens, USA

**Keywords:** mucinous adenocarcinoma of the appendix, folfiri, folfox-6, cytoreductive surgery and hipec, pseudomyxoma peritonei, appendiceal cancer

## Abstract

Primary appendiceal carcinomas are rare and often found incidentally when the appendix is surgically removed. Adenocarcinoma predominates the histological types of malignancies, with mucinous adenocarcinoma being the most prevalent of the various subtypes. Pseudomyxoma peritonei (PMP), a complication seen in mucinous adenocarcinoma of the appendix (MAA), is the collection of mucinous ascites in the intra-abdominal cavity and the thickening of the surrounding viscera by mucin-producing tumor cells. PMP initially presents with increased abdominal discomfort and girth and, in later stages, presents with obstructive abdomen symptoms. These symptoms are nonspecific and can be a challenge to pinpoint. Such was the case for our patient, in this case report, who initially presented with dyspepsia and later demonstrated compressive symptoms and weight loss, raising concern for malignancy. An appendiceal pathology was of concern when his right lower quadrant pain acutely worsened during an abdominal ultrasound, and imaging and biopsy confirmed MAA with PMP. The aim of this report is to shed light on the management of recurrent MAA. Our patient's recurrent MAA was managed with debulking procedures and three rounds of hyperthermic intraperitoneal chemotherapy (HIPEC) and was managed postoperatively with folinic acid, fluorouracil, and irinotecan (FOLFIRI) and bevacizumab, which in its totality helped achieve a progression-free survival of more than two years. We believe that cytoreduction and intraoperative chemotherapy prolong survival in patients with recurrent disease, as was the case with our patients. Our patient also demonstrated benefit as his disease stabilized after starting bevacizumab; however, more studies need to be performed at a larger scale to show a consistent relationship.

## Introduction

Appendiceal malignancies are a rare group of tumors usually found incidentally during appendectomy [1]. Adenocarcinoma is the most predominant type of primary appendix cancer, comprising 60% of all cases, and mucinous adenocarcinoma of the appendix (MAA) is the most prevalent subtype of primary adenocarcinoma with an incidence rate of 37%-38% among all appendiceal cancers [2-4]. In terms of clinical presentation, in over 50% of cases of appendiceal cancer, the patient is asymptomatic, and the malignancy is detected incidentally [1]. In 30% of cancer cases where patients present symptomatically, they present as acute appendicitis [1]. MAA management depends on the histological type and the extent of the disease at presentation [2]. For MAA that has not ruptured, the preferred management is appendectomy with great caution to prevent capsular rupture [1,2]. In this case report, we present a patient diagnosed with recurrent MAA and treated with a combination of surgery and chemotherapy. This article discusses the incidence, clinical presentation, and management of mucinous adenocarcinoma of the appendix.

## Case presentation

**Figure 1:**
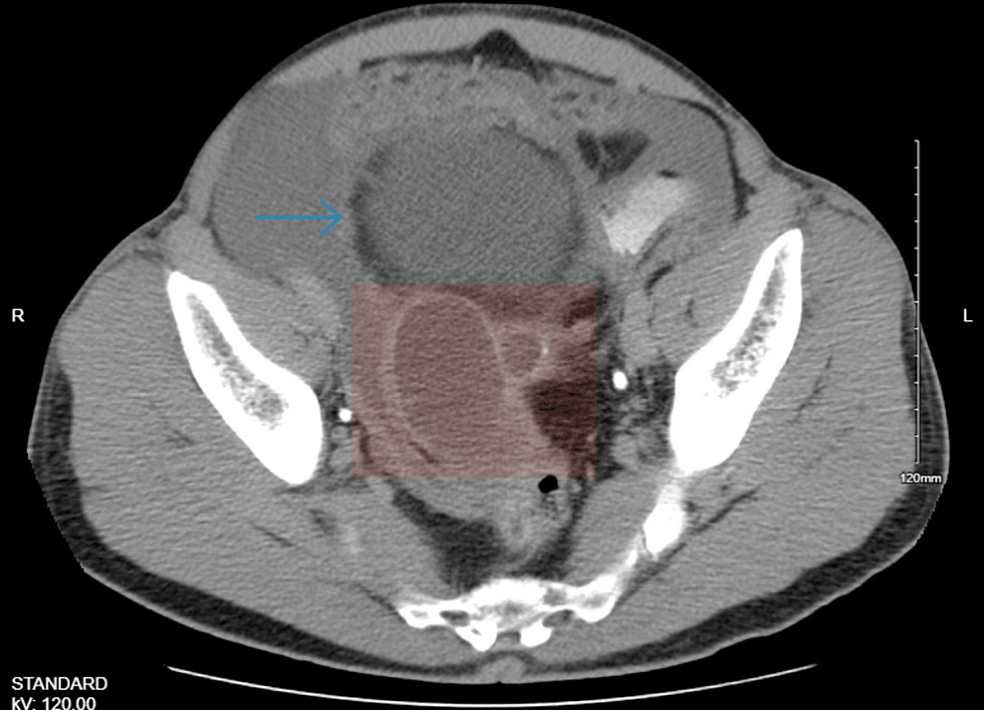
CT of the abdomen and pelvis showing a multiseptated calcified mass (shaded area) posterior to the urinary bladder (blue arrow).

The patient is a 51-year-old male who presented to the primary care physician complaining of bloating and early satiety after meals for two months. He was diagnosed with dyspepsia and managed with proton pump inhibitors. He did not experience any relief of symptoms with therapy, and he developed additional symptoms, including constipation, change in bowel habits, fecal urgency with small amounts of stools, urinary frequency, and a 7 lb weight loss over the next two months. He also developed a right lower quadrant sharp, aching pain, which waxed and waned, lasting for about one minute. An esophagogastroduodenoscopy and colonoscopy performed were unremarkable, and a repeat colonoscopy was advised in 5-7 years. During an ultrasound of the pelvis, a mass was seen in the right lower quadrant; however, before the examination could be completed, he developed severe right lower quadrant pain, for which he was referred to the emergency department and admitted. A CT scan of the abdomen and pelvis showed a large 6.4 × 4.4 × 4.4 cm multiseptated calcified mass in the right lower quadrant posterior to the urinary bladder with abdominopelvic ascites and peritoneal implants, with short-segment wall thickening in the ascending colon to the level of the hepatic flexure (Figure 1).

**Figure 2:**
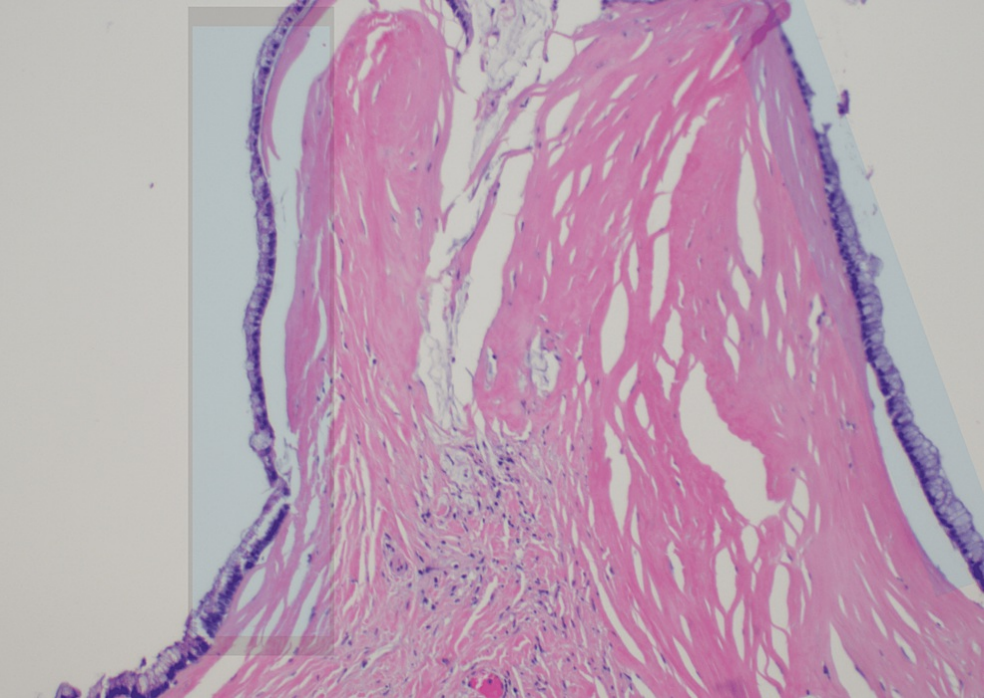
Liver nodule. The surface of the liver involved by mucinous neoplasm with mucinous deposits containing tumor cells (highlighted area), compatible with G2, moderately differentiated mucinous adenocarcinoma of appendiceal primary.

**Figure 3:**
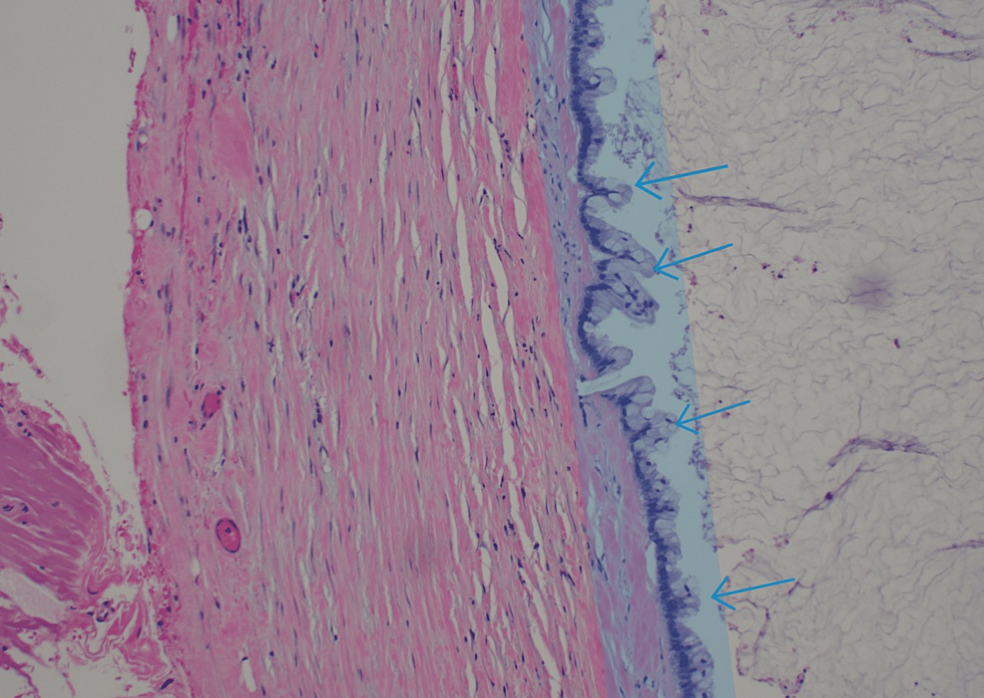
Soft tissue peritoneal implant biopsy showing mucinous neoplasm with mucinous deposits containing tumor cells.

**Figure 4:**
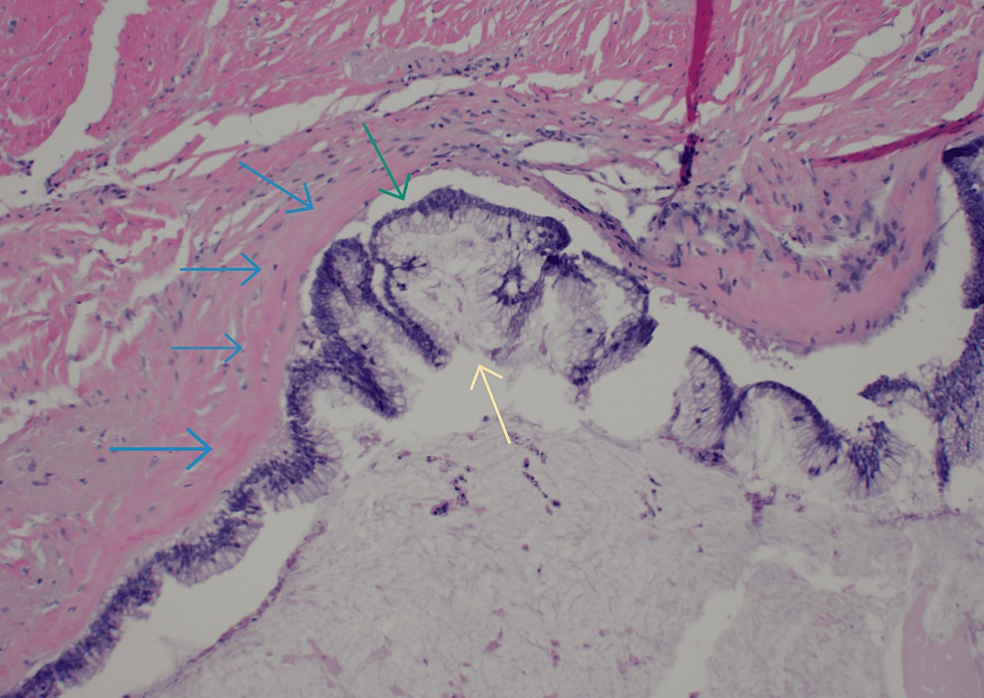
Anterior abdominal wall showing fibrous tissue (blue arrows) involved by mucinous neoplasm (yellow arrow) with mucinous deposits containing tumor cells (green arrow).

CT-guided biopsy confirmed mucinous adenocarcinoma. PET/CT scan showed a mucinous-filled tubular fluid-filled cystic appendix that was not metabolically active with low-grade uptake localizing to abdominal and pelvic ascites with peritoneal implants. Cancer antigen (CA) 19-9 levels and carcinoembryonic antigen (CEA) were 378.5 U/mL (0-37 U/mL) and 37.5 ng/mL (0-3.8 ng/mL), respectively. An exploratory laparotomy revealed omental caking and peritoneal implants, with metastases in the spleen, liver, and distal pancreas. Subtotal colectomy was performed with omentectomy, distal pancreatectomy, splenectomy, and cauterization of metastatic foci on the liver, bladder, and peritoneum. Ascitic fluid (5 L) was drained, with no residual macroscopic disease noted. Hyperthermic intraperitoneal chemotherapy (HIPEC) was delivered post-procedure for 90 minutes. Pathology slides obtained intraoperatively from a liver nodule (Figure 2), soft tissue peritoneal implant (Figure 3), and anterior abdominal wall (Figure 4) are shown.

He was enrolled in folinic acid, fluorouracil, and oxaliplatin (FOLFOX) chemotherapy with oxaliplatin removed when he developed peripheral neuropathy. He was followed at regular intervals and was able to manage work and his activities of daily living. A year later, a PET/CT scan obtained due to rising CEA on chemotherapy showed an interval increase in the disease. Therefore, a repeat exploratory laparotomy was done with cholecystectomy, porta hepatis debulking, and lesser curvature lymph node removal, which on pathology showed 100% replaced by the tumor as per pathology. Although several other lymph nodes were seen to be enlarged around the stomach, warranting near-total gastrectomy, the procedure was deemed too extensive at that time; therefore, debulking with partial gastrectomy with gastrojejunostomy with HIPEC was done. The postoperative swallow study showed no signs of a leak. He was started on capecitabine initially at 1500 mg twice a day and later reduced to 1 g once daily as he developed the hand-foot syndrome. A surveillance CT four months later showed stable disease in perigastric, gastrohepatic, perihepatic, and periportal regions. He tolerated the chemotherapy well over the next two years without any associated complications. However, his disease gradually progressed with metastatic spread to involve multiple mesenteric implants, eight years after initial diagnosis. Therefore, an impact trial was initially done, but no actionable target was found. Therefore, bevacizumab was started, capecitabine was discontinued, and folinic acid, fluorouracil, and irinotecan (FOLFIRI) was initiated. The CEA was followed, and the disease remained stable over the next year before CEA began increasing again. The case was presented to the tumor board, who agreed to another debulking surgery, which was successfully done. A subtotal gastrectomy with multiple mesenteric, peritoneal, and pelvic implants and lymph nodes was resected. After, HIPEC, gastrojejunostomy, and duodenojejunostomy were performed for reconstruction eight years after the initial diagnosis, with a total follow-up of 10 years from the initial diagnosis and two-year survival after the initiation of bevacizumab.

## Discussion

Primary appendiceal carcinomas are a rare entity. Contributing to an incidence rate of less than 0.5% of all gastrointestinal malignant neoplasms and accounting for 0.7%-1.4% of all the specimens collected after an appendectomy, they have a lower incidence rate among all digestive tract tumors [5,6]. Despite being a small vestigial organ, primary appendiceal carcinomas are composed of a wide variety of histological types, including adenocarcinoma (which are further subdivided into colonic adenocarcinoma, mucinous adenocarcinoma, and signet ring cell tumor), appendiceal neuroendocrine carcinoma, and mixed tumor containing both of these elements with goblet cells [1,2].

Adenocarcinoma is the most predominant type of primary appendix cancer, comprising 60% of all cases. With an incidence rate of 37%-38% among all appendiceal cancers reported, mucinous adenocarcinoma is the most prevalent subtype of primary adenocarcinoma with a mean age of presentation at 60 years of age, no explicit sex predilection, and no known risk factors [2-4]. Furthermore, MAA is further classified as low and high grade, which is essential in deciding the treatment options and predicting the prognosis [2].

Pseudomyxoma peritonei (PMP), first described by Karl Rokitansky and later by Werth in association with mucinous adenocarcinoma of the ovary in 1884 and Frankel in association with an appendiceal cyst tumor in 1901, is a rare malignant growth characterized by mucus-secreting tumor cells within the abdomen and pelvis. It develops due to the rupture of mucinous growth through the appendix wall leading to the spread of mucus-secreting tumor cells within the abdominal cavity, large amounts of mucinous ascites, intra-abdominal jelly collections, and the thickening of the visceral wall [7,8]. Having a slow indolent course, PMP usually presents with abdominal discomfort/pain and increasing abdominal girth. In the later stages, PMP presents as an abdominal mass and obstructive/compressive symptoms. Less than one-third of MAA presents as acute appendicitis, and rarely in males, PMP can present as an inguinal hernia [8-10]. Diagnosing MAA and PMP can be tricky, especially in the earlier stages of the disease when patients have no or less concerning symptoms that usually point toward a less concerning diagnosis, as was the case with our patient, who was initially diagnosed with dyspepsia. In the later stages of the disease, when patients present with increasing abdominal girth, compressive symptoms, and weight loss, suspicions tend to be more inclined toward malignancy, and imaging modalities come into play. Our patient had a unique presentation in the sense that he not only did present with nonspecific signs and symptoms such as bloating, early satiety, weight loss, increased abdominal girth, altered bowel movements, and urinary frequency but also developed acute right lower quadrant abdominal pain while undergoing ultrasound, alarming for an acute appendiceal pathology/emergency.

The diagnosis of MAA and PMP requires a combination of both imaging and histology. CT scan is a sensitive imaging modality to detect an underlying appendiceal malignancy and for staging. For MAA, cystic dilation of the appendix or a focal soft tissue mass is seen in the majority of cases [9]. CT scan findings suggestive of PMP include areas of low attenuation intermixed with areas of high attenuation due to solid components within the mucinous material and scalloping of the visceral surfaces, especially the liver and spleen, which distinguish PMP from fluid ascites [7]. Studies into genetic mutations that might be responsible for PMP, such as the amplification of *MCL1* and *JUN*, as well as mutations in *GNAS* and *KRAS*, not only represent novel prognostic markers but also might direct management and require further study [11]. Tumor markers, including CEA, CA 19-9, and CA 125, serve as prognostic markers and can be used for follow-up [7]. Grading and staging (the Tumor Node Metastasis {TNM} staging system) of the adenocarcinoma of the appendix are similar to the adenocarcinoma of the colon; however, MAA leading to PMP does not carry a prognosis as worse than its colonic counterpart as the appendiceal malignant cells may be noninvasive [9]. For MAA with peritoneal dissemination, measuring the extent of the disease burden is a crucial step in management. The peritoneal carcinomatosis index (PCI), as described by Jacquet and Sugarbaker [12], is the most widely used metric for assessing disease burden. PCI uses imaging modalities such as CT scans and MRI pre-operatively and produces a score that is suggestive of the extent of the disease and guides management. A score greater than 20 makes complete surgical resection challenging [2].

The management of MAA depends on the tumor size, location, histology, and whether or not the lesion has perforated. For low-grade MAA without evidence of rupture, en bloc, appendectomy is the treatment of choice and can be done via laparoscopy or open surgery, depending on the tumor size. Removing the appendix en bloc is essential to avoid the spillage of mucin and tumor cells into the peritoneal cavity. For tumors that are T2 or higher and high-grade MAA without evidence of rupture, right hemicolectomy is the procedure of choice due to the higher risk of lymph node involvement [2,13]. For patients whose diagnosis is made during appendectomy with pathological evidence of low-grade MAA and evidence of rupture, laparoscopy is recommended to determine the spread of the disease/disease burden. Patients with low-grade MAA and no gross evidence of dissemination require periodic surveillance, while patients with low-grade MAA and gross evidence of dissemination need cytoreduction. If appendectomy reveals high-grade MAA, laparoscopy or laparotomy is required to determine the disease burden. If there is gross evidence of dissemination, cytoreduction with intraperitoneal chemotherapy (particularly intraoperative hyperthermic intraperitoneal chemotherapy) is the management of choice. Though right hemicolectomy with high-grade MAA is a consideration, there are no known therapeutic benefits [2,14].

For patients presenting with an extensive, disseminated disease with evaluated disease burden on imaging, cytoreduction and intraperitoneal chemotherapy is the most optimal management strategy. This was the case with our patient, who underwent subtotal colectomy with omentectomy, distal pancreatectomy, and splenectomy, along with cauterization of metastatic foci on the liver, bladder, and peritoneum and intraoperative hyperthermic intraperitoneal chemotherapy (HIPEC) delivered post-procedure for 90 minutes. In both low- and high-grade MAA, disease recurrence is a common finding even after complete cytoreduction and intraoperative chemotherapy. Therefore, for disease recurrence, repeat cytoreduction/debulking along with intraoperative chemotherapy is a reasonable option [2]. There is minimal literature on the surgical management of recurrent disease, and we hope that this case report will help serve as a reference value for treating recurrent MAA. Our patient underwent debulking procedures and HIPEC a total of three times. We believe that cytoreduction and intraoperative chemotherapy prolong survival in patients with recurrent disease, though this topic requires further research.

Chemotherapy recommendations are pretty similar to those for colorectal cancer. Low-grade MAA is generally nonresponsive to systemic chemotherapy and hence is not recommended. For high-grade MAA and PMP, management includes the addition of systemic chemotherapy. Reduced abdominal tumor burden, fewer visceral resections, and improved progression-free survival have been associated with pre-operative systemic chemotherapy [14]. Postoperative chemotherapy has improved progression-free survival in patients with high-grade MAA treated with cytoreduction and intraperitoneal chemotherapy [15]. Fluorouracil-based chemotherapy (FOLFOX and FOLFIRI) is the most widely used chemotherapeutic regimen. In an observational study by Pietrantonio et al., the FOLFOX regimen showed promising results with a response rate of 20%, a 65% disease control rate, and a median progression-free survival of eight months [16]. Apart from FOLFOX and FOLFIRI, another accepted chemotherapeutic regimen is capecitabine with oxaliplatin (XELOX). Recently, genome-based precision medicine has made great leaps in treating various cancers. Several angiogenesis pathway factors have been identified contributing to cancer pathogenesis, one of them being vascular endothelial growth factors (VEGF). This opens the gateway for promising new research into VEGF inhibitors such as bevacizumab in treating appendiceal cancer. A meta-analysis of nine clinical trials showed strongly positive results in terms of progression-free survival (hazard ratio {HR}: 0.617; 95% confidence interval {CI}: 0.530-0.719) and overall survival (HR: 0.848; 95% CI: 0.747-0.963) favoring the bevacizumab group [17]. In a retrospective analysis, combining bevacizumab with chemotherapy helped to achieve a progression-free survival of 5.98 months and overall survival of 14.77 months [18]. The use of bevacizumab has been reported in a patient with appendiceal adenocarcinoma that helped to achieve long-term disease stabilization [19]. Its use has also been reported in a patient with recurrent MAA, helping to reduce CA 19-9 levels dramatically and pseudomyxoma on a CT scan within four months of its use [20]. In our patient, using bevacizumab helped achieve a progression-free survival of two years. Although the data is quite limited and much more research needs to be done to explore the drug's effectiveness, we hope that this case report will encourage the scientific community to consider the use of bevacizumab apart from the traditional chemotherapeutic regimen in the management of appendiceal carcinoma.

## Conclusions

In summary, we have reported a patient with a case of recurrent mucinous adenocarcinoma of the appendix that was managed with multiple surgeries and multiple different chemotherapy regimens. Our case emphasizes the importance of combining surgical management with chemotherapy to slow cancer progression, as our patient achieved a progression-free survival of two years. We hope that our case promotes further studies into the treatment of recurrent mucinous adenocarcinoma of the appendix, especially regarding the combination of cytoreduction and intraoperative chemotherapy and the use of bevacizumab.
